# Hydrophobic Modification of Layered Clays and Compatibility for Epoxy Nanocomposites

**DOI:** 10.3390/ma3042588

**Published:** 2010-04-06

**Authors:** Jiang-Jen Lin, Ying-Nan Chan, Yi-Fen Lan

**Affiliations:** 1 Institute of Polymer Science and Engineering, National Taiwan University, Taipei 10617, Taiwan; E-Mails: jan.in.nan@gmail.com (Y.C.); d95549006@ntu.edu.tw (Y.L.); 2 Department of Materials Science and Engineering, Center of Nanoscience and Nanotechnology, National Chung Hsing University, Taichung, Taiwan

**Keywords:** layered silicates, clay, epoxy, nanocomposite, compatibility

## Abstract

Recent studies on the intercalation and exfoliation of layered clays with polymeric intercalating agents involving poly(oxypropylene)-amines and the particular uses for epoxy nanocomposites are reviewed. For intercalation, counter-ionic exchange reactions of clays including cationic layered silicates and anionic Al-Mg layered double hydroxide (LDH) with polymeric organic ions afforded organoclays led to spatial interlayer expansion from 12 to 92 Å (X-ray diffraction) as well as hydrophobic property. The inorganic clays of layered structure could be modified by the poly(oxypropylene)amine-salts as the intercalating agents with molecular weights ranging from 230 to 5,000 g/mol. Furthermore, natural montmorillonite (MMT) clay could be exfoliated into thin layer silicate platelets (*ca.* 1 nm thickness) in one step by using polymeric types of exfoliating agents. Different lateral dimensions of MMT, synthetic fluorinated Mica and LDH clays had been cured into epoxy nanocomposites. The hydrophobic amine-salt modification resulting in high spacing of layered or exfoliation of individual clay platelets is the most important factor for gaining significant improvements of properties. In particular, these modified clays were reported to gain significant improvements such as reduced coefficient of thermal expansion (CTE), enhanced thermal stability, and hardness. The utilization of these layered clays for initiating the epoxy self-polymerization was also reported to have a unique compatibility between clay and organic resin matrix. However, the matrix domain lacks of covalently bonded crosslink and leads to the isolation of powder material. It is generally concluded that the hydrophobic expansion of the clay inter-gallery spacing is the crucial step for enhancing the compatibility and the ultimate preparation of the advanced epoxy materials.

## 1. Introduction

The recent development of polymer/layered silicate composites has been fruitful for industrial applications. The successful commercialization of Nylon 6/silicate clay nanocomposites by the Toyota research group [1,2], has marked the progress of utilizing mineral clays for advanced materials [3,4,5] including fire retarding and gas barrier products. Present in large quantities in nature, the smectite clays are well-characterized for their lamellar structure of multiple sheets and a large number of ionic charge sites [6,7,8]. The phyllosilicate clays of the 2:1 type including montmorillonite, bentonite, saponite, and hectorite generally consist of multiple layers of silicate/aluminum oxide with a sandwiching structure of two tetrahedron and an edge-shared octahedral sheets [9]. In the case of montmorillonite (MMT), the primary units are of multiple aluminosilicate sheets with irregular polygonal shapes at average dimension of *ca.* 100 nm × 100 nm × 1 nm for individual sheets [10]. The existed divalent counter cations in the natural clays are exchangeable with Na^+^, Cu^2+^, Zn^2+^, Mg^2+^, and Ca^2+^ ions [11]. In the order of decreasing charge density as follow, Al^3+^ > Ca^2+^ > Mg^2+^ > K^+^ = NH^4+^ > Na^+^ [12,13], indicates a possible exchange reaction with an organic quaternary ammonium salt. The intercalation of the clay layered structures with organic agents often alters the natural clays to become hydrophobic and compatible with polymers. Analogous to the natural clays, synthetic fluorine mica (Mica) is prepared from the treatment of talc with Na_2_SiF_6_ at high temperature [14,15]. Mica, conventionally used as inorganic thickener, has a considerably wider lateral dimension of 300–1000 nm in comparison with MMT (80–100 nm). Another class of synthetic clays, layered-double-hydroxides (LDH), can be prepared from the co-precipitation of inorganic salts as the represented structure, [Mg_6_Al_2_(OH)_16_]CO_3_·4H_2_O for the magnesium/aluminum hydroxides. These anionic clays may be exchanged by anionic organic species such as carboxylic acids. Other metal hydroxides including Ni, Cu, or Zn for divalent and Al, Cr, Fe, V, or Ga for trivalent metal ions, and anion such as CO_3_^2−^, Cl^−^, SO_4_^2−^, and NO_3_^−^ were reported [16,17,18,19].

The preparation of polymer/clay nanocomposites generally involves a cationic exchange reaction with organic salts such as alkyl quaternary ammonium salts [20]. For example, MMT with the structure of silicate surface (≡Si–O^−^Na^+^) can be intercalated with organic salts [21,22,23,24], including quaternary alkyl ammonium (R_4_N^+^X^−^) or alkyl phosphonium (R_4_P^+^X^−^) salts. The intercalation or organic incorporation is accompanied with a silicate gallery expansion. For example, the C_18_-alkyl quaternary salts could expand MMT interlayer spacing to 20−30 Å basal spacing from 12 Å in the original clay [25,26,27,28]. It was noticed that the same organic salt could not exchange with the clays with divalent counter ions such as Mg^2+^ or Ca^2+^ in MMT [29]. Low molecular-weight of alkyl ammonium [30,31,32,33,34,35] and phosphonium [36,37,38,39,40,41] salts are commonly used for widening basal spacing in the range of 13−50 Å [42,43,44,45].

In this review, we discuss the involvement of hydrophobic polyamines for affecting various natural and synthetic clays such as MMT, Mica, and LDH. With hydrophobic and high molecular-weight of poly(oxyalkylene)-diamines (POP-salt), the basal spacing of these clays was tailored [46,47,48,49] for their compatibilities with different epoxy systems. By varying the organics, the modified organoclays could exhibit properties of dispersing [50,51] and self-assembling properties [52,53,54]. Moreover, new developments on clay exfoliation into random individual platelets were developed [55,56]. With high surface and improved compatibilities, the clay/epoxy nanocomposites display improved physical properties, particularly in the areas of hardness, thermal stability and thermal expansion properties.

## 2. Organic Modification of Layered Clays

### 2.1. Modification of cationic sodium MMT and synthetic fluorinated Mica

A series of poly(oxyalkylene)-polyamine salts (POA-salts) including hydrophobic poly-(oxypropylene)- (POP-) and hydrophilic poly(oxyethylene)- (POE-) amines of molecular weights ranging from 230 to 5,000 g/mol had been used to modify the natural clays. As a result, high *d* spacing up to 92 Å was reported in the case of POP-amine salt of 4,000 g/mol *M*_w_ for the intercalation of Na^+^-MMT [47]. The lamellar interlayer expansion is generally proportional to molecular lengths of the intercalating agents. In the gallery of layered structures, the POP organics aggregated into a new hydrophobic phase which ultimately expanded the basal spacing. The interlayer expansion was reported to occur in a critical concentration manner similar to the so-called Critical Micelle Concentration (CMC) as a surfactant behaves in water [48]. The amount of incorporated organics was in agreement to the expanded distance of silicate interlayer spacing determined by XRD measurements [49,50].

### 2.2. Modification of anionic Al-Mg LDH

A new approach was reported for interacting with the clays through counter-ion exchange, chelating [57] and hydrogen bonding association mechanism [58] by using POA-derived amindoacids. In the chelating mechanism involving the POA-amidoacid with Na^+^-MMT, a seven-member ring cyclic intermediate was proposed. A similar mechanism with an acid-chelating intermediate was reported [30] for intercalation of alkylcarboxylic acids, CH_3_(CH_2_)_n_COOH, into the gallery of clay containing divalent metal counter ions. For comparison, the use of C_12−18_ carboxylic acids such as lauric acid (n = 10) and stearic acid (n = 16) intercalated into Na^+^-MMT resulted in only low organic embedment of 10−15 wt % organics and low XRD basal spacing of 15 Å. For the divalent M^2+^-MMT analog (K10, XRD = 10.1 Å), the same acid species could expand the silicates with a larger *d* spacing of 30 or 43 Å. The difference between the M^2+^-MMT and Na^+^-MMT intercalation was attributed to the formation of thermally stable intermediates for the divalent M^2+^, but not for Na^+^ form of MMT.

However, the LDHs are different from MMTs, not only in opposite charges of ionic characteristics but also in charge density. The strong interlayer electrostatic interaction among individual Mg-Al oxide platelets leads to a tight stacking of the lamellae and difficulty for organic incorporation. Alkyl carboxylates and sulfonates are common species as the intercalating agents, but which could widen the interlayer spacing only up to 30 Å [17,18,19]. In addition to the limitation on interlayer widening, the rate of ionic exchange reaction is considerably slow as compared to the MMT intercalation. In comparing with the alkyl carboxylic acids, the amidoacids are suitable for interacting with the anionic LDH clay. For example, POA-derived amidoacids of high molecular weight could render LDH wide basal spacing of 92 Å [59].

### 2.3. Exfoliation of MMT and Mica with multifunctional amine copolymers

Besides the wide expansion of layered silicates through intercalation, random silicate platelets could be obtained by using amphiphilic copolymers, such as Mannich condensates, hydrophobic backboned polyamidoacids and other polyamines [55,56]. The subsequent formation of amine-HCl salts was required for such exfoliation agents. Their structures generally consisting of multiple amines enabled to form stable emulsion in water with clays and exchange their counter Na^+^ ions. As a result, the layered stack of multiple silicate sheets in the Na^+^-MMT primary structure was exfoliated and randomized into individual clay platelets. The process involved the exfoliation of the layered clay through ionic exchange reaction and further NaOH treatment to phase separate and recover the organic amines. The randomized silicates were shown to have a unique ionic character and suspension in water. The physical properties of the platelets demonstrated an inherent 120 meq/100 g cation exchange capacity, averaged 720 m^2^/g surface areas, 20,000 ions, 0.9 nm^2^ area per ion and 4 × 10^16^ platelets per gram [56].

## 3. Layered Clays for Epoxy Nanocomposites 

Clay/epoxy nanocomposites have received a great deal of attentions because of their potential applications [4,5,9,13,60,61,62]. The high aspect ratio of the silicate platelets [63,64,65,66] in the polymer matrices promoted the organic materials of improved properties including mechanical strength [67,68,69,70,71], thermal stability [72], chemical resistance [73,74], and gas barrier properties [75]. In general, the process required an *in situ* monomer/clay polymerization or a direct polymer mixing at elevated temperature in order to exfoliate the layered silicates into randomized platelets. By simple mixing in the curing process, the POA modified organoclay was allowed to disperse into the epoxy matrix and demonstrated the improvements of mechanical and thermal properties [76,77,78,79]. In addition, the anionic LDH was affected by the POA-amidoacids in a similar manner for making nanocomposites. The comparison had been made for the intercalated and exfoliated clays with respect to the physical properties of nanocomposites. It was shown that the exfoliated platelets of high aspect-ratio were superior to the spherical particles of silicate oxide [78]. The geometric shape of the silicates with high aspect-ratio is the predominant for the nanocomposite properties.

### 3.1. Intercalated and exfoliated MMT clays for epoxies

**Figure 1:**
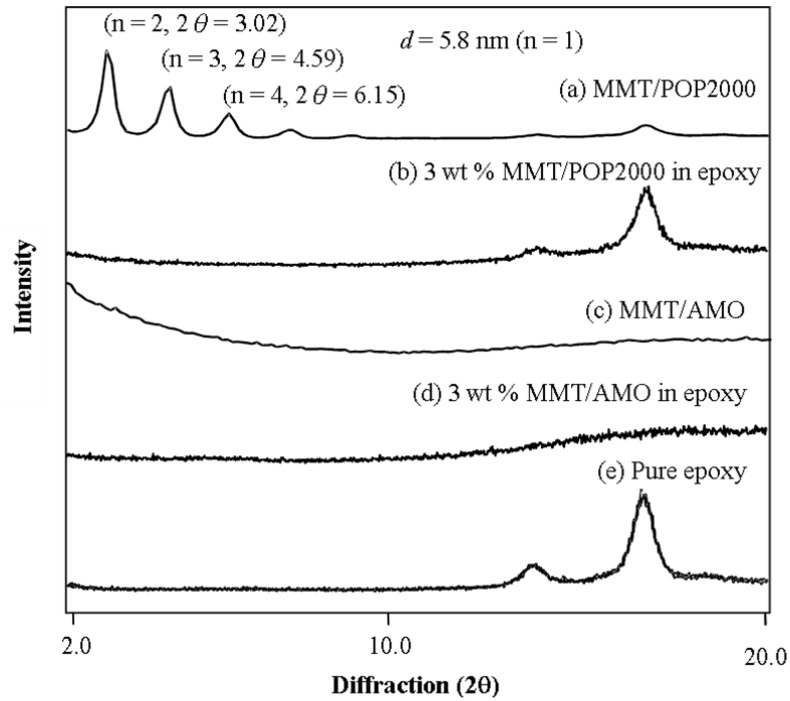
X-ray diffraction patterns of intercalated MMT/POP2000 and exfoliated MMT/AMO in cured epoxies (reprinted with permission from reference [76]).

The precursors of the intercalated and exfoliated silicates were compared for the effectiveness of affecting the epoxy matrix. The XRD technique was used to characterize the crystallographic structure of the organoclays and their dispersed homogeneity in the epoxies. As shown in Figure 1a, the XRD pattern of the intercalated MMT/POP2000 organoclay had a *d* spacing of 5.8 nm, on the basis of Bragg’s equation (nλ = 2dsinθ from the observed peaks of n = 2, 3, *etc*.) With 3 wt % loading of this organoclay to the epoxy curing system, the cured material exhibited none of the diffraction peaks in the 2–10^o^ angle (2θ) range (Figure 1b), implying the possible exfoliation of the clay layered structure. For the already exfoliated MMT/AMO organoclay (Figure 1c) as the clay precursor at 3 wt % loading, the cured materials showed no XRD peak (Figure 1d). It is noteworthy that the broad peak at 18^o^ angle (2θ) becomes amorphous, an indication of the fine dispersion of MMT platelets in disrupting the original epoxy matrix due to the presence of silicate platelets. This disruption phenomenon was observed only with the exfoliated silicates at a high loading in epoxies that had a distinct XRD peak (Figure 1e).

**Figure 2:**
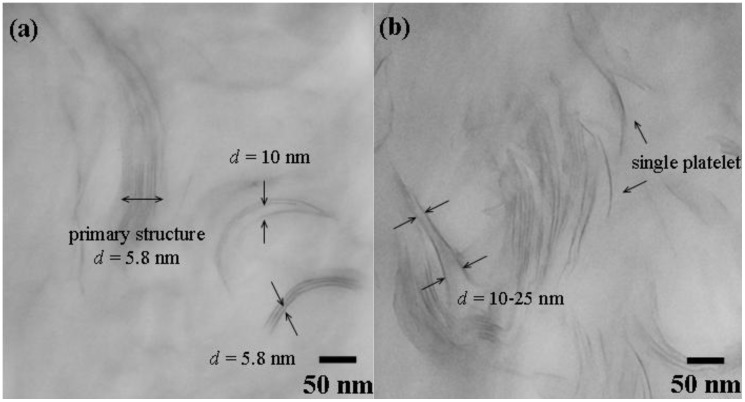
Comparative TEM micrographs of 3 wt % (a) MMT/POP2000 and (b) MMT/AMO in cured epoxies (reprinted with permission from reference [76]).

Besides XRD analyses, the dispersion of silicate platelets in epoxy matrices was analyzed by transmission electron microscopy (TEM). The layered structure of MMT/POP2000 (*d* spacing = 5.8 nm) became partial exfoliation after mixing and curing with epoxy resin, as shown in Figure 2a. Although the XRD result demonstrated a featureless pattern, some of primary stacks and the layered silicates with 2–3 layers in parallel formation remained in the epoxy nanocomposites as observed by TEM. In some cases, a three layer silicate stack of 5.8 nm spacing and layers of *ca.* 10 nm spacing remained visible in the epoxy matrices modified with the organoclay. When compared to the addition with the exfoliated clays at 3 wt % loading, much better dispersion of the platelet or homogeneous dispersion in the epoxy matrix was observed (Figure 2b). For understanding the dispersion of clay platelets in the epoxy nanocomposites, TEM is a required supplementary analysis to XRD. 

The physical properties of the epoxy materials containing the silicates were compared. In Table 1, the hardness was significantly improved from the pristine 2 H to 4–7 H, by the addition of the exfoliated silicates and the intercalated MMT/POP2000.

**Table 1:** Properties of melamine-novalac cured epoxies with the organo-MMT (reprinted with permission from reference [76]).

Organoclay	MMT content (wt %) ^a^	Hardness (H)	Transparency (%) ^b^	CTE (μm/m °C) ^c^	*T_g_* (°C) ^d^
None	0	2	60.0	66.9	113.3
MMT/POP2000	0.5	4	59.3	52.0	137.9
(XRD = 58 Å)	1	5	58.2	33.5	136.2
MMT/POP copolymer	0.5	6	57.5	47.0	140.4
(XRD, featureless)	1	7	56.1	37.5	138.8

^a^ Calculated on the basis of silicates. ^b^ Determined by using an UV-vis at 550 nm. ^c^ CTE: coefficient of thermal expansion, determined by TMA. ^d^ Based on DSC measurement.

The coefficient of thermal expansion (CTE) was correspondingly reduced at 0.5–1 wt % of clay additions, while their glass transition temperatures (*T*_g_) increased significantly from the pure epoxy 113.3 °C to 140.4 °C. The *T*_g_ increase was leveled off over 0.5 wt % loading and reminded the same for a higher loading. The leveling was explained by the excessive incorporation of low-molecular-weight organics into the organoclay compositions.

### 3.2. Comparison of platelet size of the exfoliated clay for epoxies

**Scheme 1:**
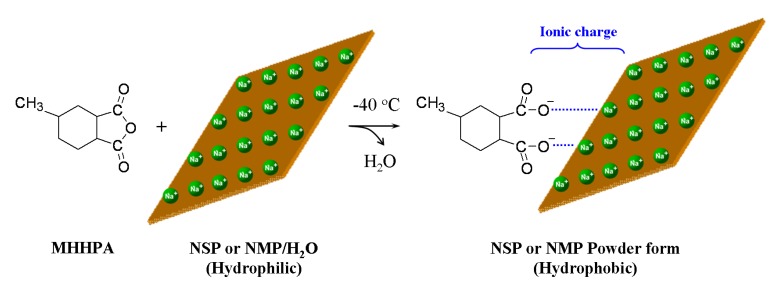
Conceptual illustration of reacting ionic silicate platelets with alkyl anhydride (reprinted with permission from reference [77]).

**Figure 3:**
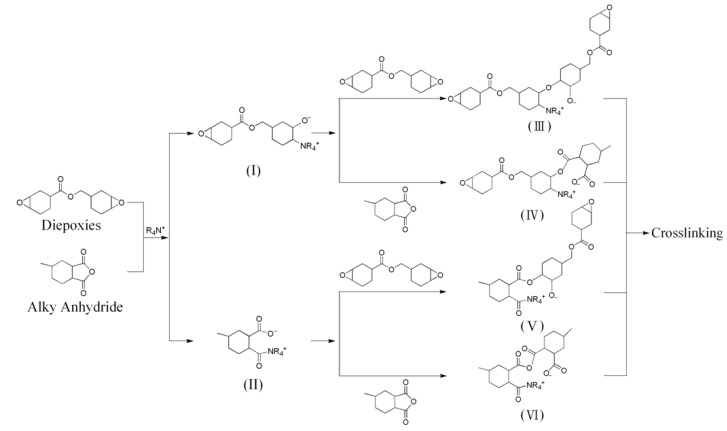
Curing mechanism of anhydride and aliphatic epoxy resin with an amine-salt catalyst (reprinted with permission from reference [77]).

The differences of the exfoliated platelets for affecting the nanocomposites are further demonstrated in the epoxies involving anhydride curing system. The platelet dimensions are polydispersed in the range of 80–100 nm for the natural Na^+^-MMT and 300–1,000 nm for synthetic Mica [10,52]. The exfoliation of MMT and Mica by using POP-derived copolymers led to the subsequent isolation of the randomized platelets in water suspensions [56,57]. Both the nanosilicate platelets (NSP) and nano- Mica platelets (NMP) were characterized as thin-platelet shapes with a thickness of 1 nm, but with different width dimensions, 80–100 nm and 300–1000 nm, for NSP and NMP, respectively. These inorganic silicate platelets are hydrophilic and dispersible in water owing to the presence of sodium ions (≡Si−O^−^Na^+^) on the platelet surface. The organic modification with alkyl anhydride through the ionic complexing on the platelet altered the materials to be hydrophobic. The alkyl anhydride (methyl hexahydrophthalic anhydride or MHHPA) has been chosen because of its common use as an epoxy agent in the two-component epoxy system. The anhydride modification for the platelets with ionic charges had been proposed (Scheme 1). The two components of epoxy curing with alkyl anhydride were well documented in literatures [76,77,78]. The curing mechanism involving the reaction of anhydride-epoxy ring-opening polymerization is complicated [78,79]. According to the reaction scheme of Figure 3, two types of polymer chain propagation were proposed. With di-functional epoxies, cross-linking polymerization occurred.

**Figure 4:**
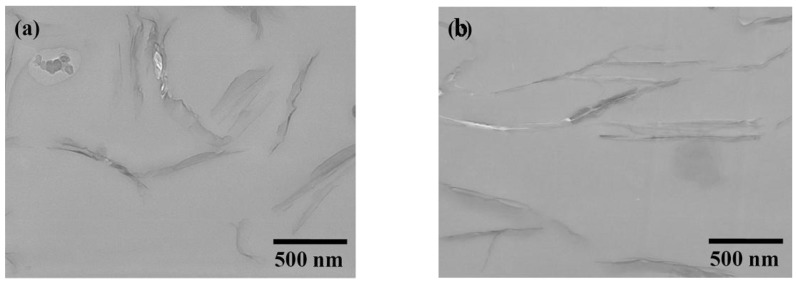
TEM of silicate platelet alignments in cured epoxies; (a) NSP and (b) NMP (reprinted with permission from reference [77])

**Scheme 2:**
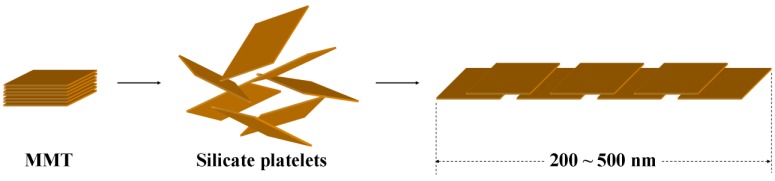
Conceptual description of the exfoliated platelets undergoing a secondary alignment.

With the involvement of NSP and NMP through the anhydride modification, different loadings of 0, 0.1, 0.3, 0.5, and 0.7 wt % demonstrated the NSP/epoxy and NMP/epoxy nanocomposites were compared. The epoxy materials showed a low CTE and high hardness while maintaining the transparency. The TEM directly showed the morphologies of the NSP and NMP in epoxy composite (Figure 4), as homogeneously embedded in the anhydride-cured epoxy matrix. However, the platelets tended to form a secondary alignment with a lamellar or lengthy bilayer formation. The lamellar orientation may extend its continuous alignment to a length of 200−500 nm for NSP and 300−1,000 nm for NMP. The formation of the lamellae in the epoxy matrix may well be reflected the nature of ionic character of the inorganic platelets, and eventually contributes to the physical performance of the nanocomposite. The platelet self-alignment is conceptually described in Scheme 2.

For the enhanced properties, the NSP and NMP effect was compared in Table 2. By adding 0.5 wt % of nanosilicates, CTE was significantly lowered from 82 μm/m °C to 37 μm/m °C for NSP and to 36 μm/m °C for NMP. The decrease in CTE, limited at 0.5 wt % loading, was caused by the intensive interaction of platelet surface with organic epoxies. Consistently, the hardness was improved from 4 to 7 H for NSP and to 8 H for NMP. By using an optical UV-vis absorption technique, the material transparency was measured. The maintained transparency of 90 % implied a fine dispersion of the platelets in the matrices without a serious light interference.

**Table 2:** Properties of anhydride-cured aliphatic epoxy nanocomposites with silicate platelets (reprinted with permission from reference [77]).

Silicate platelets	Loading (wt %) ^a^	CTE (μm/m °C) ^b^	Hardness (H) ^c^	Transparency (%) ^d^
None	0	82	4	85
NSP	0.1	78	4	84
	0.5	37	7	77
NMP	0.1	71	4	84
	0.5	36	8	80

^a^ Calculated on the basis of silicates. ^b^ CTE: coefficient of thermal expansion, determined by TMA. ^c^ Pencil test. ^d^ Determined by using a UV-vis absorption method at 550 nm/T %.

### 3.3. Comparison of spherical silica and silicate platelets for epoxie

**Figure 5:**
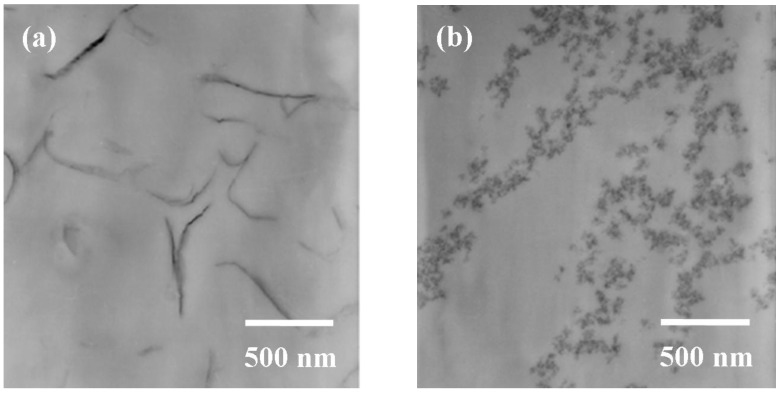
TEM of cured epoxies containing 3 wt % of (a) silicate platelets (b) silicate oxide particles (reprinted with permission from reference [78]).

The commercially available spherical shape silicate oxide was compared with clay silicate platelets with 80−100 nm dimensions. The comparison showed, in the amine curing epoxies, that both hardness and *T_g_* were enhanced. By TEM (Figure 5), the silicate platelets appeared to be in a bilayer arrangement with a length of 200−500 nm. By comparison, the silicate particles appeared to be aggregated in clusters.

In Table 3, the hardness increases from the pristine 2H to 2−3 H and 4 H by loading 1 and 5 wt % of particle silicate are shown. By comparison, an increase to 5 H with a lesser amount of platelet silicate was obtained. However, no significant difference in CTE decrease was observed. The inconsistent data for the CTE trend between Table 2 and Table 3 are noticeable. It is also realized that there data are based on different epoxy systems, melamine-novalac, anhydride and amine curing agents for Table 1, Table 2 and Table 3, respectively. Secondly, in Table 3 using amine curing, the hardness failed to increase as high as that in Table 2 (up to 8H) while the transparency was poor in Table 3. It appears the comparison indicates the silicate platelets in Table 3, suffered from a less satisfactory degree of dispersion. The dispersion method was improved in a later publication [77] for those experiments cited in Table 2.

**Table 3:** Properties of amine-cured epoxy with silica oxides and silicate platelets (reprinted with permission from reference [78]).

Silicates	Loading (%)	CTE (μm/m °C) ^a^	Hardness ^b^ (H)	Transparency ^c^ (%)
None	0	98	2	61
SiO_2_	1	80	3	54
SiO_2_	5	72	4	45
Silicate platelets	0.5	90	4	55
Silicate platelets	1	95	5	48

^a^ CTE: coefficient of thermal expansion, determined by TMA. ^b^ Pencil test. ^c^ Determined by using a UV-vis absorption method at 550 nm/T %.

### 3.4. Nanocomposites from one-component curing with organically modified LDH, MMT and Mica

**Figure 6:**
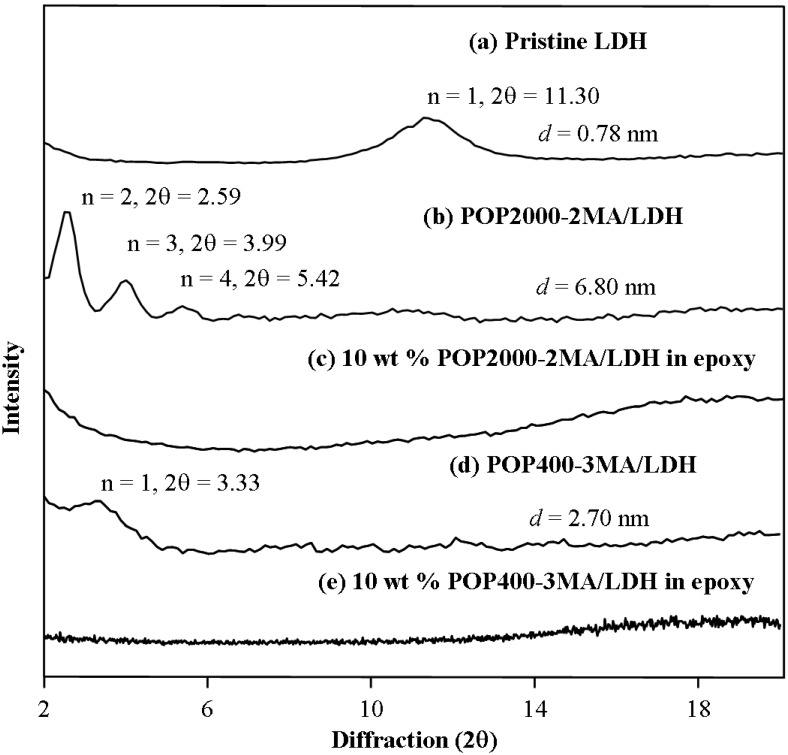
X-ray diffraction patterns of organo-LDH initiated epoxy self-curing material (reprinted with permission from reference [79]).

Organically modified Al-Mg LDHs were prepared by the intercalation of POP-acids (400 and 2,000 *M*_w_) [79]. The organo-LDHs were used to initiate the self-curing of the epoxy resins without adding a second curing agent component. The organo-LDHs with POP2000- and POP400-acid modification (6.80 nm and 2.70 nm spacing, respectively) were cured into epoxy materials showing featureless XRD diffraction patterns (Figure 6, showing the comparison of LDH, organo-LDH and the cured epoxies with 10 % of clay). Furthermore, the TEM observation for the 10 wt % loading of the organo-LDH demonstrated the LDH platelet distribution in a homogeneous manner in the epoxy matrix.

**Figure 7:**
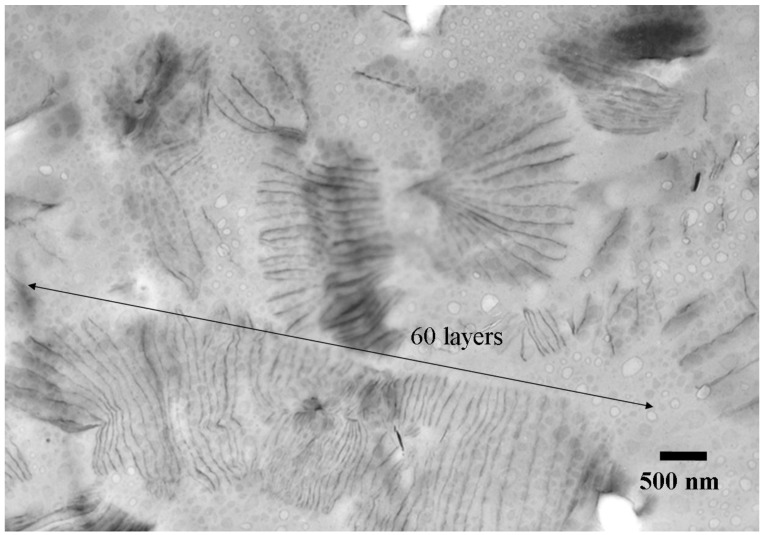
TEM of 3 wt % Mica/POP2000 initiated epoxy self-curing material (reprinted with permission from reference [80]).

With the use of organo-MMT or -Mica for initiating epoxy one-component curing [80], the reaction occurred differently from the organo-LDH. With 3 wt % of Mica/POP2000 loading, the epoxy resin was self-polymerized into a powder material rather than the solid form of fully cured epoxies as in the amine or anhydride two-component systems. The step-wise curing process under the condition of 80 °C, 150 °C and 180 °C occurred exothermically. The resultant powder was analyzed by XRD, TEM and SEM, showing a featureless reflective pattern and large interlayer spacing, as shown in Figure 7. The TEM images showed Mica platelet sizes of 1,000 nm and interlayer spacing between two neighboring platelets is quite large (120–610 nm) or mostly 500 nm. The long-range distance was over 10 nm or beyond the detection limit of XRD. Furthermore, a greater than 60 layer parallel formation with a considerable regularity was seen in the micrographs.

## 4. Conclusions

Recent developments utilizing natural clays that require an organic modification through intercalation and exfoliation with polymeric amine-salts have been discussed. The modified clays became compatible with hydrophobic epoxy resins and suitable for curing into nanocomposites. Both intercalated and exfoliated clays have been produced using polyamine salts and further made into two-component (DGEBA and amine) cured nanocomposites. The POP-modified MMT, Mica, and LDH clays were compared for enhancement of the epoxy properties of hardness and lower CTE. In particular, the exfoliated platelets could remarkably enhance the hardness from 2H to 6H in comparison with the intercalated clays of 4 H.

For the epoxy one-component or self-cured nanocomposites, the spatially expanded organo-LDH, -MMT and -Mica could act as the initiators for the epoxy ring-opening polymerization, Perhaps due to the lack of cross-link formation, the resulting material assumed a powder form with very high platelet spacing (120–610 nm) rather than the typical solid appearance.

**Figure 8:**
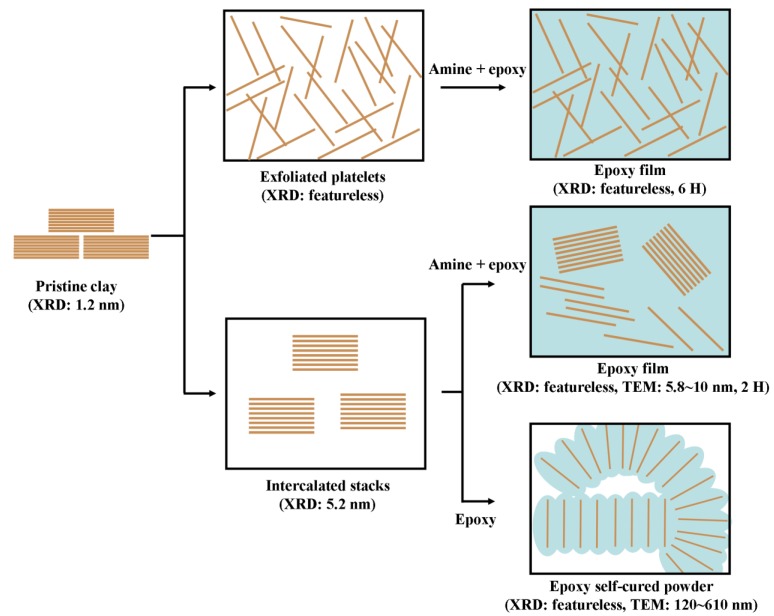
Conceptual illustration of two types of amine-cured epoxy films and clay-initiated epoxy powder.

As conceptually illustrated for the differences in organic modification (Figure 8), two-component epoxies required exfoliated platelets more than intercalated stacks for enhancing their final material hardness. However, the intercalated silicates can disperse well in one-component epoxy with the epoxy monomer diffusion into the clay intra-gallery and proceeded self-polymerization. It is noted the organic modification on clay markedly makes the difference in compatibility and hence their advanced properties of the epoxy nanocomposites.
